# Clinical features and inflammatory signatures of patients with persistent gastrointestinal long COVID two years after severe SARS-CoV-2 infection

**DOI:** 10.1038/s41598-026-37595-8

**Published:** 2026-01-29

**Authors:** Arlene dos Santos Pinto, Victor Irungu Mwangi, Juliana Costa Ferreira Neves, Alex Bezerra Silva Maciel, Alexandre V. Neto, Jefferson da Silva Valente, Gisely Cardoso de Melo, Wuelton Marcelo Monteiro, Vanderson de Souza Sampaio, Allyson Guimarães da Costa, Fernando F. Almeida-Val

**Affiliations:** 1 Programa de Pós-Graduação em Medicina Tropical (PPGMT), Universidade do Estado Do Amazonas, Manaus, 69040-000 AM Brazil; 2 Fundação de Medicina Tropical Dr. Heitor Veira Dourado, Manaus, 69040-000 AM Brazil; 3 Diretoria de Ensino e Pesquisa, Fundação Hospitalar de Hematologia e Hemoterapia do Amazonas (HEMOAM), Manaus, 69050-001 AM Brazil; 4 Programa de Pós-graduação em Imunologia Básica e Aplicada (PPGIBA), Universidade Federal do Amazonas, Manaus, 69080-900 AM Brazil; 5 Instituto Todos Pela Saúde, São Paulo, 01310-942 SP Brazil; 6 Universidade Federal do Amazonas, Manaus, 69077-000 AM Brazil

**Keywords:** Long COVID, Gastrointestinal symptoms, Inflammation, SARS-CoV-2, Brazil, COVID-19, Diseases, Gastroenterology, Immunology, Medical research

## Abstract

Persistent gastrointestinal (GI) symptoms are increasingly recognized as part of long COVID, yet their underlying mechanisms remain poorly defined. We conducted an exploratory case-series study of 80 adults hospitalized with severe COVID-19 in March-May 2020 in Manaus, Brazil. Two years post-infection, participants underwent structured clinical interviews and longitudinal cytokine analysis (IL-1β, IL-6, IL-8, IL-10, IL-12, and TNF-α). Overall, 30 participants reported ongoing GI symptoms (GI group) predominantly gastroesophageal reflux (63%), abdominal pain (43%), and diarrhea (37%). Compared with participants without GI symptoms (nGI group, *n* = 50), the GI group reported a higher burden of additional long COVID symptoms, including palpitations, headache, and arthralgia. They also exhibited distinct clinical and laboratory features, including lower baseline creatinine and ferritin levels and altered platelet indices. Although IL-6 levels were lower during the acute hospitalization phase, they became significantly elevated at four months post-infection (D120, *p* = 0.005), suggesting delayed inflammatory response. Ascendent biomarker analysis identified TNF-α as highly expressed in a large proportion of GI group. The findings suggest GI problems can persist two years after severe COVID-19, and long-term inflammatory dysregulation may underlie the pathogenesis of these GI manifestations in long COVID. Prolonged gastrointestinal surveillance in COVID-19 survivors is necessary.

## Introduction

Since the onset of the COVID-19 pandemic, increasing attention has turned to its long-term consequences, known as post-COVID-19 condition or long COVID^1^. Characterized by persistent or new symptoms lasting more than 12 weeks after acute infection, long COVID affects a wide range of systems^1–3^. While fatigue, respiratory complaints, and neurocognitive symptoms are among the most commonly reported^3,4^, gastrointestinal (GI) manifestations, such as abdominal pain, diarrhea, gastroesophageal reflux, and dysphagia, have also emerged as frequent and disabling outcomes^5,6^. A post-hoc analysis from a prospective multi-center cohort study reported a 4.5% prevalence of post-COVID-19 gastrointestinal disease. The patients from this group experienced worsening abdominal pain, hunger pain, heartburn, and acid regurgitation, unlike the improvement or stability of symptoms observed in controls without gastrointestinal disease. It was noted that COVID-19 infection is associated with the development of newly diagnosed disorders of gut-brain interactions and distinct symptom trajectories when compared with patients with pre-existing irritable bowel syndrome/functional dyspepsia^7^. Another large cohort study involving Korean patients observed an exponential increase in incidence of gastroesophageal reflux disease (GERD; HR1.36; 95% CI 1.18–1.56) and functional bowel disorders (HR 1.21; 95% CI 1.00–1.47) during the post-acute phase of SARS-CoV-2 infection among individuals with acute SARS-CoV-2 infection than in uninfected individuals^8^.

Evidence from large cohort studies in the USA and UK suggests that GI symptoms can persist for months or even years following SARS-CoV-2 infection, regardless of the severity of the initial illness^9,10^. Large cohort studies have demonstrated increased risk for functional GI disorders post-COVID-19, even among non-hospitalized individuals^9,11^. However, the mechanisms driving these symptoms remain poorly defined. Potential contributors include direct viral entry via ACE2 receptors in intestinal epithelial cells, gut microbiota disruption, immune dysregulation, and chronic low-grade inflammation^12–15^.

Cytokines such as interleukin-6 (IL-6) and tumor necrosis factor-alpha (TNF-α) have been implicated in both acute COVID-19 severity and in the pathophysiology of long COVID^16,17^. Despite increasing recognition of long COVID, few studies have explored their temporal profiles in relation to long-term gastrointestinal outcomes. Notably there’s a paucity in studies evaluating long-term gastrointestinal sequelae in underrepresented populations with severe early-pandemic infection such as that in the Brazilian Amazon. Moreover, most existing research has been conducted in high-income countries, with limited data from low-resource settings where the burden of disease and healthcare inequities may affect both exposure and recovery. In Latin America, Manaus city, the capital of Amazonas state, Brazil, experienced a unique first-wave exposure profile, which saw the collapse of basic healthcare system due to the high rates of infections and deaths. Given the high transmission and incidence, and a mixed-income society, such a setting provided suitable cohort of COVID-19 survivors. This population provides an opportunity to link acute-phase clinical and cytokine data to post-acute outcomes.

We conducted a prospective cohort study in Manaus, Brazil, an early severely impacted COVID-19 epicenter in Latin America. The study’s objective was to evaluate the prevalence of persistent gastrointestinal symptoms (two years after hospitalization) in long COVID, and to investigate their associations with demographic factors, clinical characteristics, laboratory findings, and circulating inflammatory cytokine levels over time.

## Results

### Demographic, clinical characteristics and prevalence at two years

**Fig. 1:**
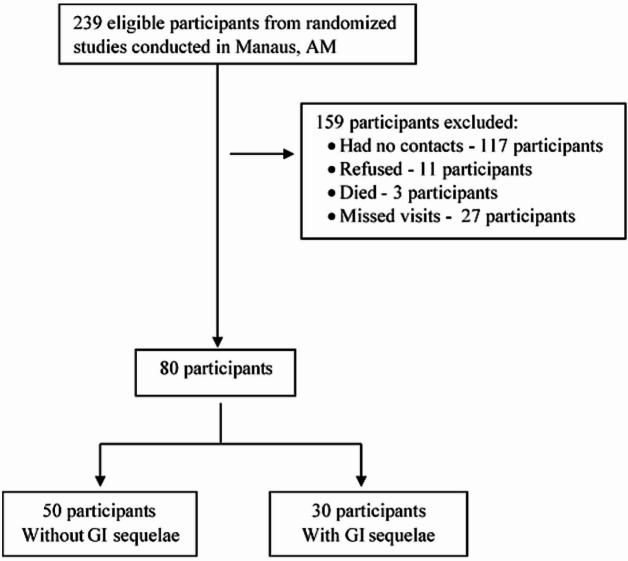
Flowchart illustrating selection of the 80 participants.

Of the 239 patients hospitalized for COVID-19 during the first pandemic wave in Manaus, 80 were selected and included in the present study (Fig. 1). The mean age of participants with GI symptoms was 52 years, and most (70%) were female. There were significant differences in sex and racial distribution between the two groups (*p* = 0.037 and *p* = 0.002, respectively). There was no remarkable difference in the length of hospital stay or ICU admission rates between the two groups (Table 1). At enrolment into the study, palpitations, alopecia, headache, arthralgia, and cough (all *p* < 0.05) were significantly more common in the GI group (those with persistent gastrointestinal symptoms at two years follow-up) compared to the participants without the post-COVID-19 GI symptoms (Table 1).

**Table 1:** Laboratory profile of the population studied on at baseline.

Variables	GI group	nGI group	*p*-value ^2^
	N = *30* ^*1*^	N = *50* ^*1*^	
Age, years (SD)	52 (14)	53 (14)	0.5
Sex			
Feminine	21/30 (70%)	23/50 (46%)	0.037
Masculine	9/30 (30%)	27/50 (54%)	
Race			
White people	9/30 (30%)	4/50 (8%)	0.002
Mixed race	16/30 (53%)	43/50 (86%)	
Black people	2/30 (7%)	3/50 (6%)	
East Asian	3/30 (10%)	0	
Weight, Kg (SD)	84 (19)	81 (19)	0.5
Height, cm (SD)	161 (9)	163 (10)	0.3
BMI, kg/m^2^ (SD)	32.4 (7.5)	30.3 (5.6)	0.3
Has comorbidities, (%)	26/30 (87%)	46/50 (92%)	0.5
Chronic heart disease (%)	0	2/46 (4%)	-
Chronic lung disease (%)	1/26 (4%)	3/46 (7%)	> 0.9
Previous tuberculosis (%)	0	1/45 (2%)	-
Diabetes mellitus (%)	7/26 (27%)	11/46 (24%)	0.8
Liver disease (%)	6/26 (23%)	3/46 (7%)	0.063
Chronic neurological disease (%)	5/26 (19%)	4/46 (9%)	0.3
Obesity (%)	16/26 (62%)	24/46 (52%)	0.4
Smoking habits			
Yes	1/26 (4%)	0	0.5
Never smoked	18/26 (69%)	32/46 (70%)	
Ex-smoker	7/26 (27%)	14/46 (30%)	
Alcoholism			
Yes	5/26 (19%)	13/46 (28%)	0.7
No	16/26 (62%)	26/46 (57%)	
Ex-alcoholic	5/26 (19%)	7/46 (15%)	
Abdominal pain, yes (%)	20/30 (67%)	23/50 (46%)	0.073
Vomiting, yes (%)	12/30 (40%)	15/50 (30%)	0.4
Nausea, yes (%)	20/29 (69%)	25/50 (50%)	0.1
Arrhythmia, yes (%)	1 (3%)	0	0.4
Chest pain, yes (%)	16 (53%)	6 (12%)	< 0.001
Heart failure, yes (%)	2 (7%)	1 (2%)	0.6
Palpitation, yes (%)	18 (60%)	13 (26%)	0.003
Alopecia, yes (%)	21 (70%)	17 (34%)	0.002
Skin rash, yes (%)	1 (3%)	2 (4%)	> 0.9
Fatigue, yes (%)	24 (80%)	27 (54%)	0.019
Headache, yes (%)	17 (57%)	10 (20%)	< 0.001
Arthralgia, yes (%)	18 (60%)	16 (32%)	0.014
Muscle weakness, yes (%)	2 (7%)	2 (4.0%)	0.6
Cough, yes (%)	16 (53%)	11 (22%)	0.004
Odynophagia, yes (%)	0 (0%)	1 (2%)	> 0.9
Nasal congestion, yes (%)	1 (3.3%)	1 (2%)	> 0.9
Wheezing, yes (%)	1 (3.3%)	0	0.4
Anosmia, yes (%)	2 (6.7%)	4 (8%)	> 0.9
Patient hospitalized, yes (%)	23/30 (76.67%)	41/50 (82%)	0.6
Admitted to ICU, yes (%)	2/23 (8.70%)	4/41 (10%)	> 0.9
Intubated, yes (%)	2/23 (8.70%)	4/41 (10%)	> 0.9

GI group: those with persistent gastrointestinal symptoms at two years; nGI group: those without gastrointestinal symptoms at two years follow-up; BMI: Body mass index; Kg: kilogram; cm: centimeter; SD: Standard deviation; ICU: Intensive care unit.

^*1*^ *Mean (SD); n/N (%);* ^*2*^ *Wilcoxon rank-sum test; Wilcoxon exact rank-sum test; Fisher’s exact test*.

By day 720 (D720) of follow-up, the GI symptoms reported by the GI group were gastroesophageal reflux (63%), abdominal pain (43%), diarrhea (37%), and dysphagia (10%). Over half of the affected participants presented with more than one GI symptom.

### Profile inflammatory cytokines and overall biomarker signatures

**Fig. 2:**
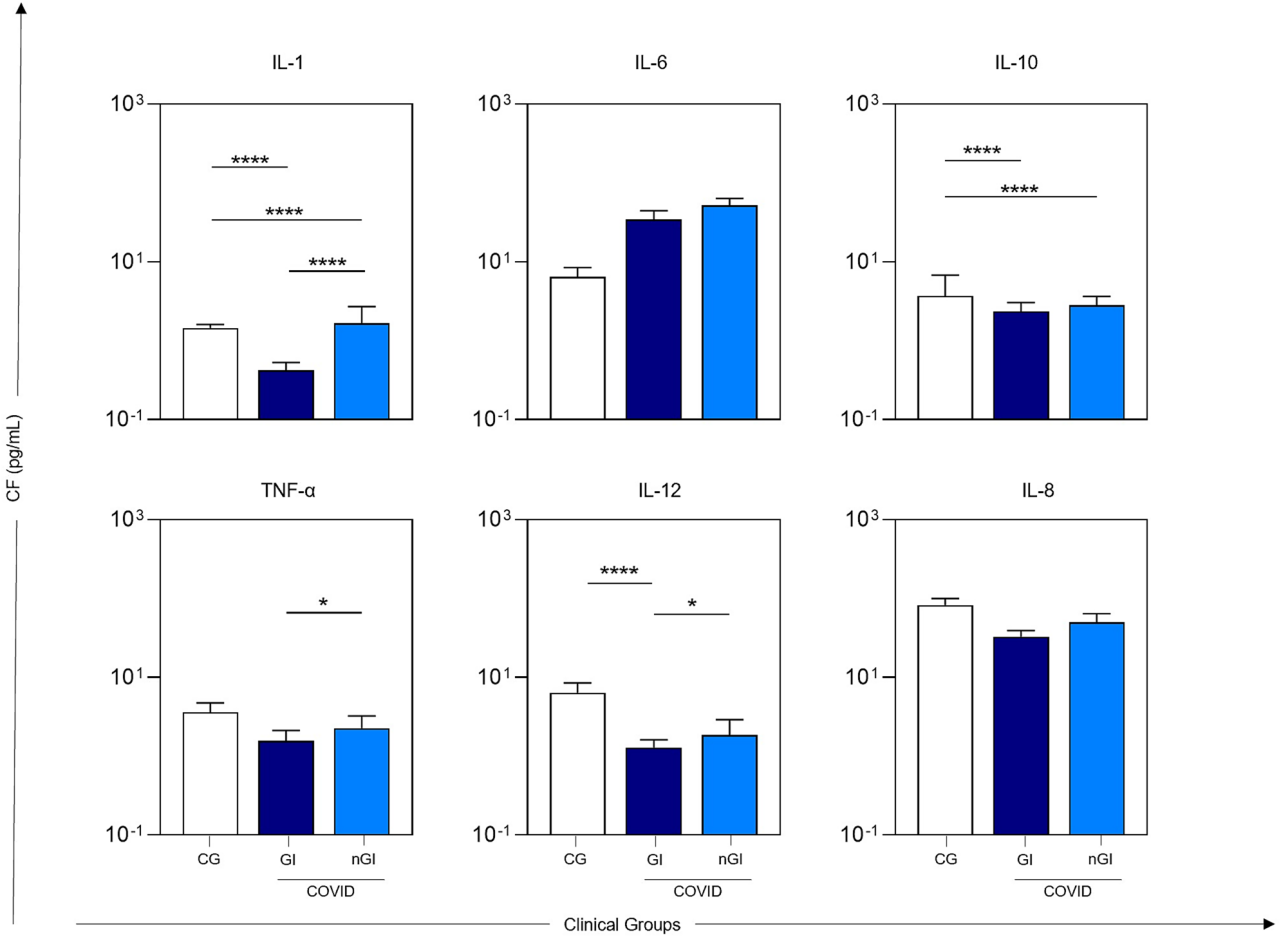
Analysis of six immunological mediators at D1, showing cytokine concentration profiles (pg/mL, log scale) across three groups: healthy controls (CG square filled with white colour) COVID-19 patients with gastrointestinal sequelae (GI square filled with dark blue colour), and COVID-19 patients without persistent GI symptoms (nGI square filled with sky blue colour). GI group: those with persistent gastrointestinal symptoms at two years; nGI group: those without gastrointestinal symptoms at two years follow-up.

To further elucidate immunological differences between the groups, baseline cytokine profiles were compared (Fig. 2). Levels of IL-1 and IL-12 were significantly lower in the GI group compared to those of the non-gastrointestinal (nGI) and control groups. Additionally, IL-6 levels were reduced in both GI and nGI groups relative to controls. Notably, TNF-α levels were significantly lower in the GI group than the nGI group, but no remarkable changes in the IL-8 profile (Fig. 2).

**Fig. 3:**
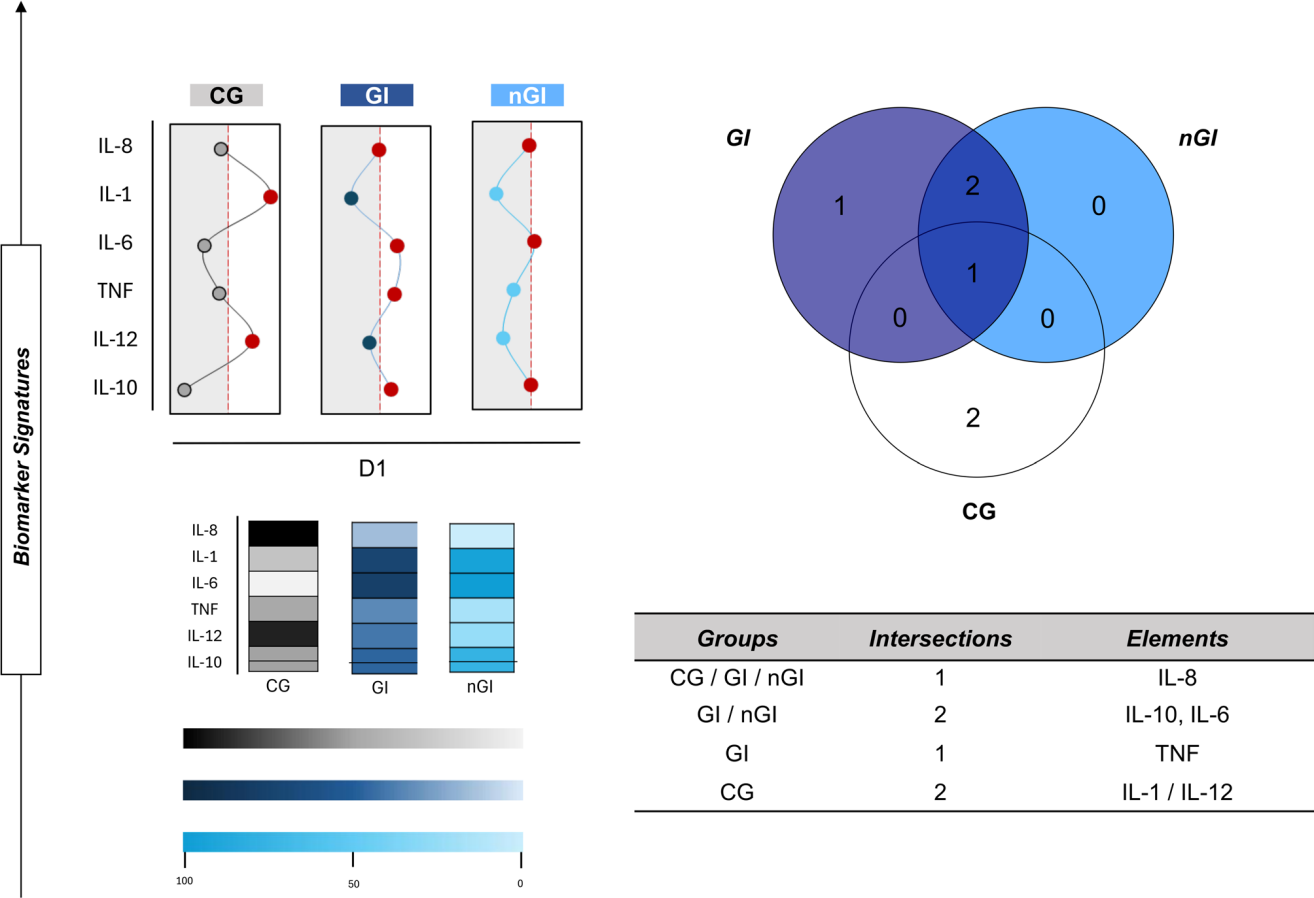
Ascendant signature of soluble immunological molecules in GI and nGI patients at D1. The analysis was based on the global median levels of each measured cytokines across all participants including healthy controls (CG), without persistent GI symptoms (nGI), and those with persistent GI symptoms (GI). The global median, expressed as mean fluorescence intensity (MFI), was used as the cutoff to classify individuals as “low” or “high” producers of each molecule. Statistical significance was set at *p* < 0.05. The Venn diagram highlights molecules uniquely elevated in the GI group, suggesting their potential as biomarkers of persistent GI symptoms.

To determine the potential biomarkers specific to the GI patients, we conducted additional biomarker signature analysis. This approach allowed for the identification of molecules considered highly produced, defined as those exceeding 50% global median of that immunological molecules or biomarker in the three distinct study groups: CG, GI, and nGI. The graph at D1, in Fig. 3, illustrates the likely biomarker signatures for each group, where the red dashed line represents the 50% global median threshold; molecules whose points lie to the right of this line are classified as high producers. The CG group presented high levels of IL-8 and IL-1, while the nGI group also demonstrated high production of IL-8 and IL-1. On the other hand, the GI group stands out for its high production of IL-10 and IL-6 (Fig. 3).

To complement the visualization, the color map at the bottom of the graph provides a scale of molecular production intensity. The color gradation, from gray/dark blue to light blue, denotes an increase in molecule production, with darker shades corresponding to higher levels, that is, above the 50% threshold of the global median. In summary, this analysis allowed the identification of molecules produced in high quantities by each group, revealing both overlaps and exclusivity in production, with TNF emerging as a distinctive marker for the nGI group.

In addition, a venn diagram details the overlap and exclusivity of these molecules. Interleukin 8 (IL-8) was identified as a molecule common to all three groups (CG, GI, and nGI). IL-10 and IL-6 were observed as common high producers in both the GI and nGI groups. Notably, Tumor Necrosis Factor (TNF) was a high producer exclusive to the GI group, suggesting that only this group exhibits significantly elevated TNF production compared to the others. Multivariable models did not identify age, sex, race, or comorbidities as significant predictors of persistent gastrointestinal status at two years within this cohort.

### Laboratory biomarkers, inflammatory status and cytokine profiles over time

Laboratory and biomarker analyses revealed significant alterations in immune mediator profiles and clinical indices at two years, including lower ferritin, creatinine, and platelet indices among GI patients (Table 2). These results suggest ongoing physiological or inflammatory differences between the two groups, even two years after the acute infection.

**Table 2:** Comparison of the clinical and laboratory profiles at D1 and D720 for the populations studied.

Parameters (SI units)	D1			D720		
	GI group N = *30* ^*1*^	nGI group N = *50* ^*1*^	*p*-value ^2^	GI group N = *30* ^*1*^	nGI group N = *50* ^*1*^	*p*-value ^2^
Hemoglobin (g/dL)	11.95 (1.77)	12.33 (1.55)	0.3	14.28 (1.40)	14.60 (1.60)	0.6
Leukocytes (mm^3^)	9.7 (4.3)	10.9 (4.5)	0.15	7.94 (2.13)	7.24 (2.59)	0.3
Lymphocytes (µL)	17 (10)	15 (9)	0.3	31 (8)	32 (8)	0.7
Neutrophils (µL)	74 (17)	78 (10)	0.5	59 (9)	56 (9)	0.4
Hematocrit (%)	38.7 (5.0)	40.6 (9.0)	0.5	43.4 (4.4)	43.7 (7.5)	0.7
Platelets (mm^3^)	325 (141)	293 (119)	0.2	259 (85)	218 (72)	0.014
Hemoglobin glycated (%)	ND	ND	ND	6.54 (1.27)	7.01 (2.21)	> 0.9
ALT (U/L)	54 (56)	67 (52)	0.089	33 (21)	34 (22)	0.5
AST (U/L)	36 (21)	52 (34)	0.026	23 (10)	26 (11)	0.3
Bilirubin direct (mg/dL)	0.28 (0.49)	0.26 (0.30)	0.7	0.14 (0.09)	0.16 (0.07)	0.4
Bilirubin indirect (mg/dL)	0.35 (0.34)	0.29 (0.21)	0.8	0.48 (0.28)	0.47 (0.23)	> 0.9
Bilirubin (mg/dL)	0.63 (0.81)	0.56 (0.45)	> 0.9	0.62 (0.33)	0.63 (0.26)	0.5
Glucose (mg/dL)	184 (79)	175 (56)	> 0.9	115 (38)	129 (65)	0.8
Cholesterol (mg/dL)	409 (715)	162 (35)	0.5	223 (49)	214 (53)	0.3
HDL (mg/dL)	66 (79)	65 (92)	0.6	45 (11)	45 (12)	0.7
LDL (mg/dL)	ND	ND	ND	133 (37)	123 (39)	0.12
Triglycerides (mg/dL)	258 (175)	214 (123)	0.8	214 (156)	218 (153)	> 0.9
Creatinine (mg/dL)	0.73 (0.28)	1.06 (0.66)	0.009	0.85 (0.33)	0.96 (0.25)	0.006
Urea (mg/dL)	27 (10)	38 (22)	0.007	33 (11)	35 (13)	0.5
LDH (U/L)	440 (416)	709 (440)	0.025	ND	ND	ND
CK (U/L)	166 (224)	362 (1,387)	0.9	ND	ND	ND
CK-MB (U/L)	21 (12)	21 (11)	0.8	ND	ND	ND
Phosphatase alkaline (U/L)	84 (50)	137 (42)	0.7	ND	ND	ND
Ferritin (ng/dL)	694 (782)	826 (773)	0.14	135 (130)	220 (168)	0.015
D-dimer (ng/dL)	1133 (1795)	1311 (1683)	0.6	ND	ND	ND
PCR (mg/dL)	57 (48)	70 (49)	0.5	7.60 (3.66)	8.29 (4.38)	0.4
Sodium (mEq/L)	140.4 (4.6)	140.2 (3.9)	0.6	139.3 (9.9)	137.0 (3.2)	0.5
Potassium (mEq/L)	4.08 (0.31)	4.28 (0.50)	0.07	4.30 (0.50)	4.41 (0.54)	0.3
Patient hospitalized, yes (%)	23/30 (77%)	41/50 (82%)	0.6	ND	ND	ND
Admitted to ICU	2/23 (9%)	4/41 (10%)	> 0.9	ND	ND	ND
Intubated	2/23 (9%)	4/41 (10%)	> 0.9	ND	ND	ND

GI group: those with persistent gastrointestinal symptoms at two years; nGI group: those without gastrointestinal symptoms at two years follow-up; BMI: Body mass index; LDL: Low-density lipoproteins; HDL: High-density lipoproteins; LDH: Lactate dehydrogenase; ICU: Intensive care unit; CRP: C-reactive protein; CKMB: Creatine kinase MB; AST: Aspartate transaminase; ALT: Alanine transaminase; ICU: Intensive care unit; ND: Not determined or tested.

^*1*^ *Mean (SD); n/N (%);* ^*2*^ *Wilcoxon rank-sum test; Wilcoxon exact rank-sum test; Fisher’s exact test*.

### Inflammatory cytokines longitudinal analysis

**Fig. 4:**
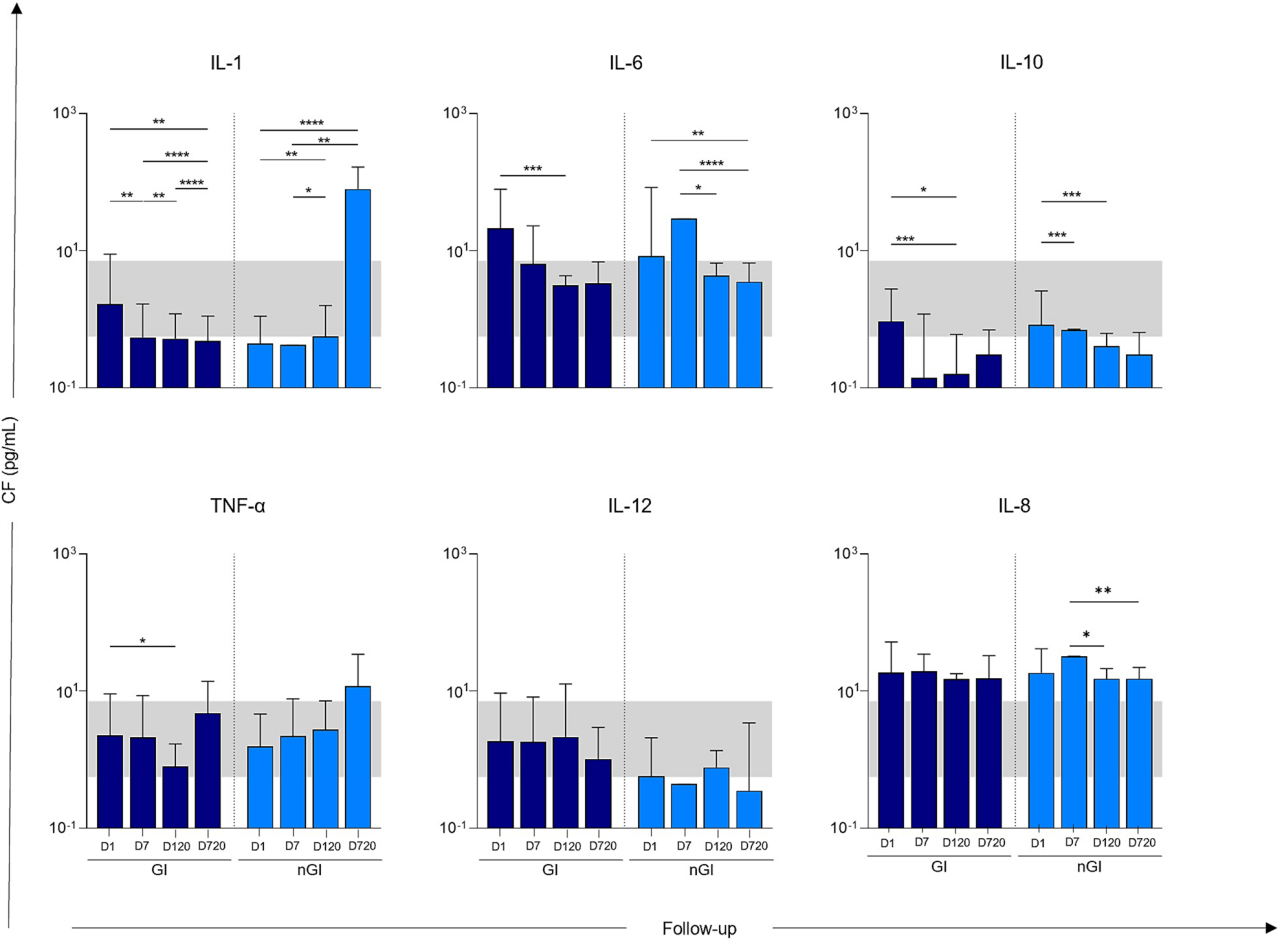
Analysis of the dynamics of soluble immunological molecule production during the clinical evolution of patients (Follow up) at days 1 (D1), 7 (D7), 120 (D120), and 720 (D720). The within-group longitudinal comparisons among individuals with gastrointestinal infection (GI square filled with dark blue colour), and those without gastrointestinal infection (nGI square filled with sky blue colour) using Friedman test, followed by Dunn’s post-hoc test. Horizontal lines indicate statistically significant differences between groups or time points (*p < 0.05, **p < 0.01, ***p < 0.001). Grey shaded areas represent cytokine levels in healthy donors (CG square filled with white colour). Error bars represent standard deviations.

A longitudinal analysis of cytokine levels revealed distinct inflammatory profiles between groups, as shown in Fig. 4. In the early acute phase (D1), inflammatory cytokines IL-1, IL-6, and IL-8 were elevated in both groups with (GI) and without (nGI) persistent GI symptoms, particularly at D1 and D7, but declined progressively over time. Among the pro-inflammatory cytokines measured, IL-1 showed a gradual increase within the GI group, with significantly higher levels at D1 which declined to D720, while an opposite trend is seen in the nGI group where IL-1 at D720 was significantly high than at baseline. At baseline, IL-6 in both groups was high at the acute phase which declined marginally by D720. While IL-10 levels in the nGI group showed a steady decline up to D720, the GI group exhibited an initial decrease from D1 to D120, followed by a modest increase by D720.

For TNF-α, a modest but significant decrease was observed in GI patients between D1 and D120 (*p < 0.05), while nGI patients showed a gradual upward trend over time, though not statistically significant. Levels of IL-12 remained consistently below the healthy control range in both COVID-19 groups, with no significant differences across time points. As for IL-8, there was an elevated level of the cytokine by D7 in the nGI group which decreased significantly by D120 and D720 (*p < 0.05 to **p < 0.01), while it remained stable in the GI group throughout follow-up.

Overall, these results suggest a prolonged alteration in cytokine profiles in both GI and nGI COVID-19 groups, with the nGI group demonstrating a more pronounced late inflammatory response (IL-1, IL-6, IL-8), and both groups showing a consistent decline in anti-inflammatory IL-10 and IL-12 levels. There are thus long-term immune changes in COVID-19 survivors, with some differences depending on GI involvement during acute infection.

### GI patients display a dysregulated inflammatory cytokine network

**Fig. 5:**
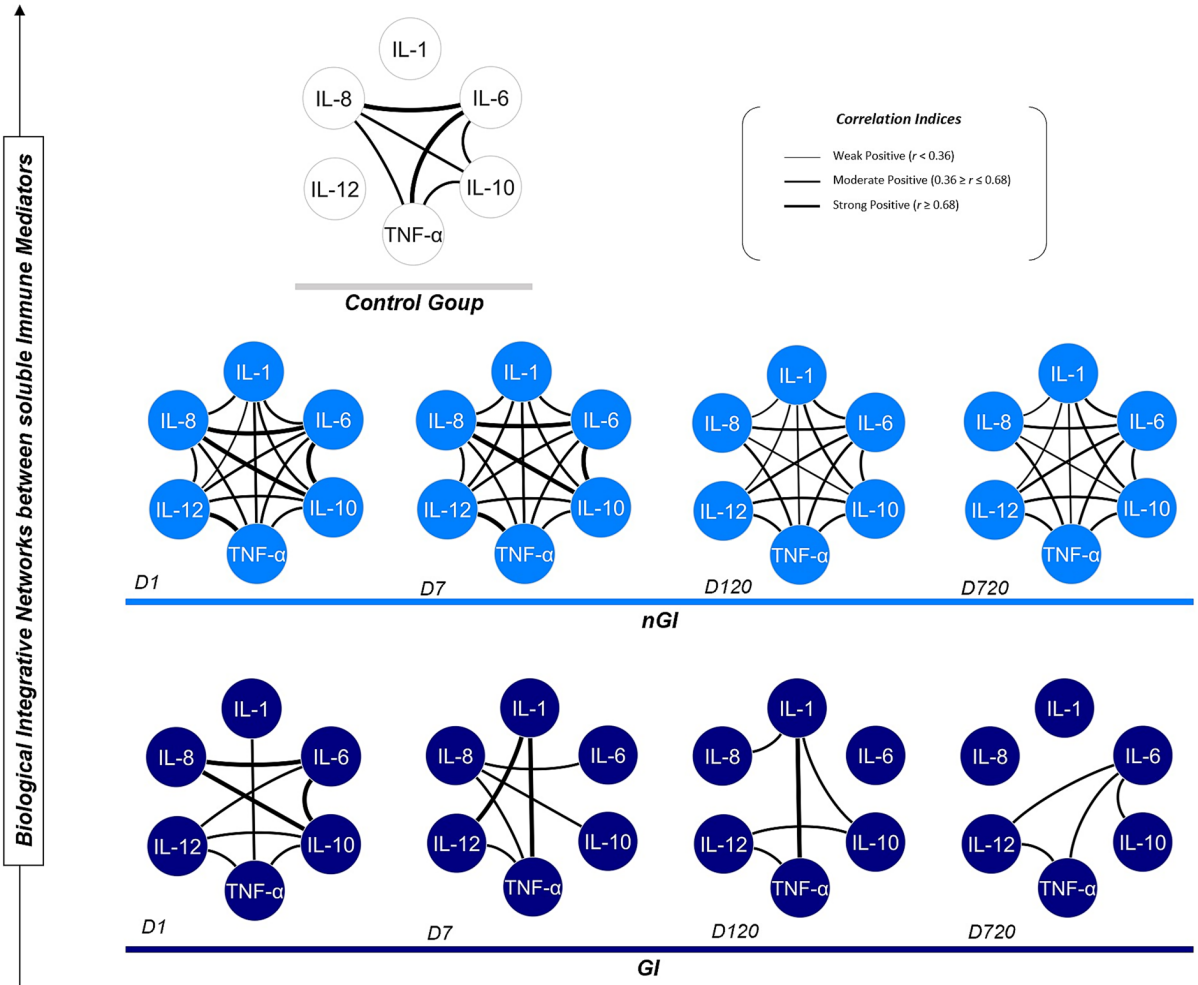
Correlation networks of soluble immune mediators (cytokines) at different time points (D1, D7, D120, D720) compared to healthy donors (CG). The thickness and type of connecting lines indicate correlation strength and direction (positive or negative). Solid lines mark positive correlations (weak to strong); dashed lines are negative correlations, with varying thickness for strength.

Correlation network analysis of cytokine profiles across time points revealed distinct immunological patterns between groups (Fig. 5). In the control group, moderate (*r* = 0.36 to 0.68) to strong (*r* ≥ 0.68) correlations predominantly involved IL-6, IL-10, TNF-α and IL-8. Among nGI patients, numerous moderate to strong correlations between the six cytokines persisted from the acute phase through to the post-COVID-19 phase, reflecting a relatively stable immune profile over time. Contrastingly, the GI group exhibited a progressive disintegration of cytokine correlations, among group particularly D120 and D720. At D1, the GI group displayed limited correlations involving IL-1, IL-8, IL-12 and TNF-α, in contrast to the more interconnected network seen in nGI patients. A similar pattern was observed on D7 for IL-10, IL-6, IL-12 and IL-1. By D720, only IL-6 maintained moderately strong correlations with IL-10, IL-12 and TNF-α in the GI group, in contrast to the cytokine network observed in the nGI group. Notably, no negative correlations were detected observed in either group.

The moderately strong correlations in the nGI patients at D720 may suggest a trend toward a more organized or stabilized cytokine interaction profile during long-term recovery from acute inflammation, but do not constitute evidence of immune reprogramming. Overall, the correlation network analysis suggests a progressive loss of immune coordination in GI patients, potentially reflecting ongoing dysregulation.

**Fig. 6:**
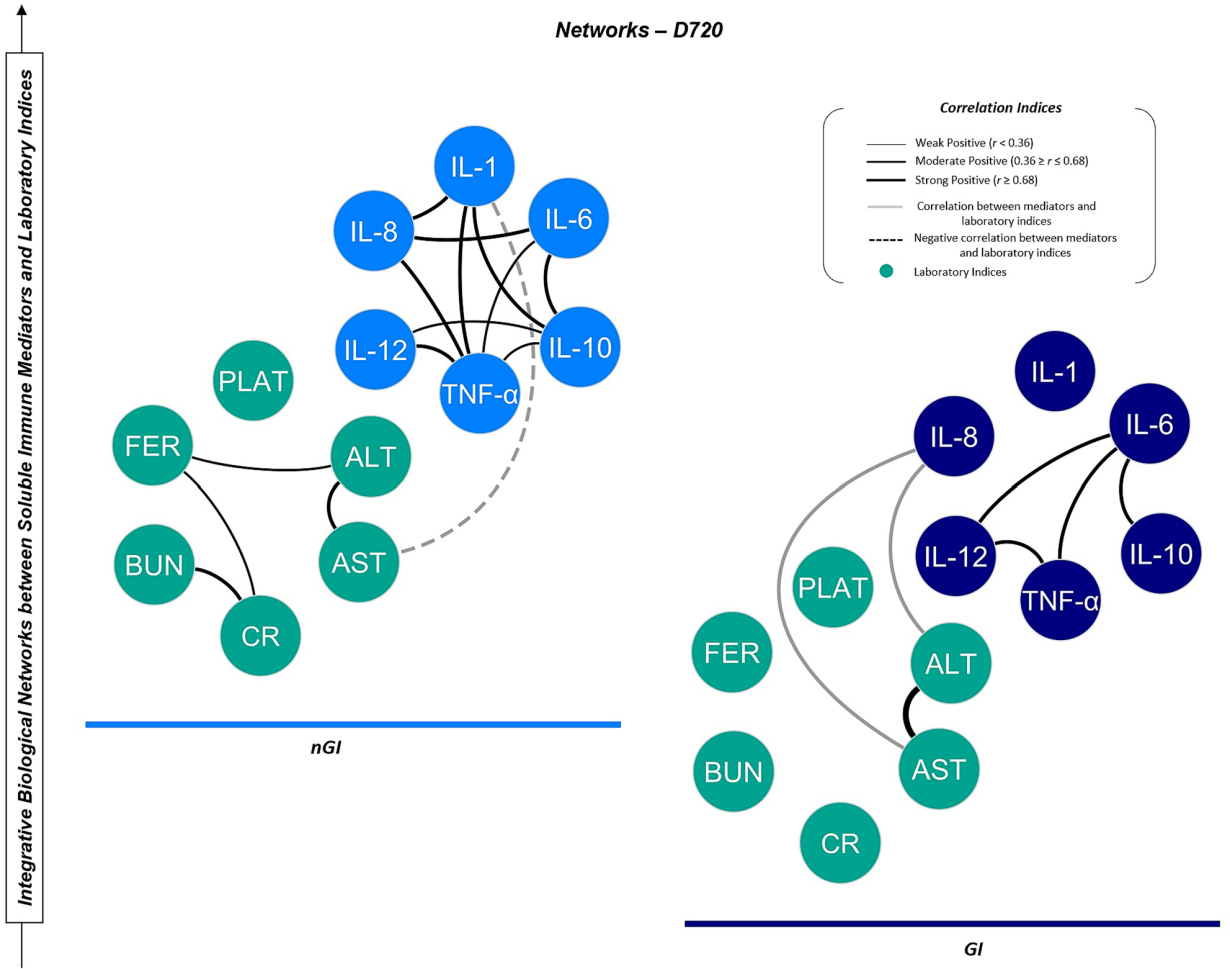
Integrative network correlation analysis of Soluble Immune Mediators and Laboratory Indices. Nodes represent inflammatory cytokines (GI group - dark blue; nGI group - light blue) and clinical laboratory markers (teal). Solid black lines indicate significant positive correlations (weak to strong); dashed lines are negative correlations, with varying thickness for strength.

An interaction network analysis between inflammatory and laboratory parameters was also explored at D720 (Fig. 6). Within the GI group, there was significant interplay between IL-8 and the liver injury markers AST and ALT, suggesting there is the link between inflammatory response and end-organ parameters, and particularly liver involvement. Notably, markers such as IL-1, ferritin, and indicators of renal function (urea and creatinine) did not demonstrate significant connectivity in this network, pointing towards a more specific pathophysiological pathway.

In contrast, there are highly interconnected networks of pro-inflammatory cytokines in the nGI group. There is also a notable interplay between ferritin, and the indicators of renal and liver functions. There is, however, an inverse relationship between systemic inflammation (IL-1β) and liver injury (AST) suggesting a regulatory role of IL-1β under certain conditions.

## Discussion

Our study presents one of the longest follow-up assessments (720 days) of hospitalized COVID-19 survivors in the Brazilian Amazon. By linking acute-phase clinical and cytokine data collected during the first pandemic wave with longitudinal inflammatory trajectories, we were able to relate delayed immune responses to long-term gastrointestinal outcomes. This study expands long COVID research beyond descriptive symptom prevalence by examining associations in a population rarely represented in existing cohorts. To our knowledge, it is the first study in the Brazilian Amazon to evaluate persistent GI symptoms alongside longitudinal systemic inflammatory profiles, and one of the few globally to examine how delayed cytokine trajectories relate to organ-specific long COVID manifestations.

In this exploratory, case-series study with a two-year follow-up, we found that the Brazilian Amazon patients, hospitalized, pre-vaccine COVID-19 patients reported persistent gastrointestinal (GI) symptoms, including reflux, abdominal pain, diarrhea, and dysphagia. These symptoms could be associated with specific inflammatory and laboratory markers, as well as the presence of other long COVID manifestations. Our findings support the growing evidence that SARS-CoV-2 infection can lead to sustained alterations in gastrointestinal physiology and immune regulation. Likewise, it corroborated with other observations of women being more frequently affected by long COVID than men^16,18^.

Recent large-scale studies have demonstrated that persistent GI symptoms are among the most common long-term manifestations of COVID-19. Xu et al. (2023), using data from the U.S. Department of Veterans Affairs, identified increased risks of gastroesophageal reflux, dyspepsia, functional intestinal disorders, and pancreatitis up to one year after infection, even in non-hospitalized individuals^6^. Similarly, our findings show that hospitalized patients may continue to experience a significant burden of GI symptoms even two years after infection, reinforcing the need for gastrointestinal surveillance in post-COVID care.

One of the most notable findings of this study is the association between persistent GI symptoms and inflammatory markers. Patients with persistent GI symptoms had distinct laboratory profiles 2 years post infection. This included lower ferritin and creatinine levels, and higher platelet counts, suggesting potential low-grade inflammation or immune dysregulation. More importantly, cytokine analyses revealed a unique temporal profile: IL-6 levels were lower during the acute phase in the GI group but became significantly elevated at four months. This exacerbated and persistent increase in IL-6, a central cytokine in COVID-19 pathogenesis, may reflect a prolonged inflammatory state contributing to the persistence of GI symptoms^6^. While IL-1β and TNF-α levels were not significantly different, trends toward altered profiles support the hypothesis of a dysregulated immune response in these individuals, which has been shown before^16,17,19^. A study by Kwon et al., demonstrated a significantly high levels of IL-1β and IL-6, alongside IL-10, VEGF, IL-8, MIP-1α, MCP-1, granzyme A, and IL-9 in participants with gastrointestinal symptoms than in those without gastrointestinal symptoms^20^. Furthermore, our signature biomarker analysis at baseline identified elevated TNF-α as unique to the GI group, which is consistent with a possible role as an early biomarker of persistent GI symptoms in long COVID and possibly associated with chronic GI symptoms. Our findings align with and reinforce previous findings reporting elevated IL-6 and TNF-α levels in association with systemic inflammation, including GI symptoms among patients with long COVID^16,21^.

A growing body of evidence supports the notion that SARS-CoV-2 induces persistent immune activation long after viral clearance, and IL-6 appears to play a central role in this process. In the gastrointestinal tract, SARS-CoV-2 targets ACE2-expressing enterocytes, potentially disrupting epithelial integrity and promoting a pro-inflammatory environment that persists beyond the acute phase. Network analysis from our study suggests that IL-6 may contribute to immune dysregulation in long COVID patients reporting chronic GI symptoms two years after infection. Mechanistically, IL-6 contributes to both local and systemic inflammation through cis- and trans-signaling pathways, adversely affecting endothelial function, epithelial barrier permeability, and mucosal immune responses^16,22–25^. Conversely, among the uninfected healthy controls and recovered COVID-19 patients, the pro-inflammatory action of IL-6 has been balanced by other equally active cytokines like IL-10, IL-8, and IL-12. For instance, cytokine interrelations likely influenced the inverse but relationship between IL-1 and AST among the nGI patients by D720. While this relationship appears paradoxical, it raises the hypothesis that suppressed IL-1 levels might play a protective role against liver injury under specific physiological conditions. This is supported by the dual nature of the IL-1 family; as noted by Barbier et al., while certain members are characteristically pro-inflammatory and drive tissue damage, others possess anti-inflammatory properties that may promote tissue regeneration and limit inflammation^26,27^. Further mechanistic studies are required to determine whether the IL-1 signaling observed here functions as a protective response or a marker of liver injury recovery. Contrastingly, IL-8 positive correlations with liver function enzymes among our long COVID patients suggests a sustained hepatic inflammation even at 2 years post-acute-phase^28–31^. Furthermore, it is possible that medications used to manage the GI symptoms could contribute to the observed liver dysfunction, though specific pharmacological intervention exposure data were not available to confirm this.

In addition, COVID-19–associated gut dysbiosis may reduce levels of anti-inflammatory metabolites like butyrate and isoDCA, further exacerbating mucosal inflammation and perpetuating IL-6 expression^5^. The cross talk between the gut and immune system, including the lung-gut axis, reinforces the systemic nature of this process. Our findings are consistent with the hypothesis that persistent low-grade inflammation, possibly sustained by altered cytokine regulation and microbial imbalance, underlies long-term gastrointestinal manifestations of COVID-19 ^6,9,32^. COVID-19 infection in the gut typically results in increased recruitment and activation of immune cells^33^. Elsewhere, a study on patients with long COVID show persistent dysregulation of a wide range of cytokines that are often associated with elevated levels of pro-inflammatory cytokines, including TNF-α^34^.

Moreover, the strong association between persistent GI symptoms and other long COVID symptoms, such as palpitations, headache, and arthralgia, suggests a multisystemic process rather than an isolated gastrointestinal condition. This aligns with previous studies showing that long COVID is characterized by overlapping symptom clusters across respiratory, neurological, and gastrointestinal domains^35–41^.

It is important to note that this study focused on hospitalized patients, most of whom experienced moderate to critical disease severity. In addition, all cases of acute COVID-19 in this cohort occurred prior to the availability of vaccines, during the initial wave of the pandemic in early 2020. The absence of vaccine-induced immune priming may have potentially influenced the trajectory of post-acute sequelae, as shown before^2,42,43^. The association between disease severity and long-term sequelae has been well documented, and it is plausible that patients with more severe initial presentations are more susceptible to lasting GI damage^44–46^. Nonetheless, long COVID has also been observed in mild and non-hospitalized cases, implying that additional factors, such as host genetics, microbiota composition, and pre-existing conditions, may influence long-term outcomes^45,47–51^. Although not assessed in this study, persistent viral reservoirs in the gastrointestinal tract and ongoing fecal viral shedding may also sustain low-grade inflammation, and dysbiosis, thus prolonged GI manifestations.

This study had several limitations. Firstly, the sample size, while adequate for exploration analysis, limits the statistical power and generalizability of the findings. Secondly, gastrointestinal symptoms were assessed through self-report at the 720-day follow-up, which may introduce recall bias or underreporting. Adding to this, some loss to follow-up occurred during the two-year interval between hospitalization and the D720 assessment, largely due to difficulty re-establishing contact, refusal to participate and missed clinical visits, and a few post-acute mortalities. As a result, our findings represent may underestimate the true burden and depth of long-term persistent GI symptoms, given that individuals who may have experienced more severe persistent GI symptoms complications did not return for evaluation (lost to follow-up). Its impact on feasibility-based long COVID cohort designs should be considered when interpreting prevalence estimates. Thirdly, the study’s analysis was limited by its reliance on peripheral blood for cytokine measurements, omitting direct mucosal assessments of gut immunity. Furthermore, neither fecal viral RNA nor microbiome assessments were conducted. Finally, the absence of clinical laboratory data from matched, COVID-19 naïve healthy controls limited our ability to contextualize the Day 720 laboratory findings in the GI group. Since the participants originated from two randomized clinical trials conducted during the first pandemic wave, the cohort consisted primarily of patients with severe acute disease who received protocolized interventions, including methylprednisolone or chloroquine. This may have influenced baseline inflammatory profiles and long-term outcomes, and limits generalizability to non-hospitalized, vaccinated, or variant-infected populations. Importantly, all participants in this cohort were infected during the first wave of the pandemic, prior to the introduction of COVID-19 vaccines. As such, the findings may not be generalizable to individuals who experienced breakthrough infections post-vaccination or to more recent viral variants. Furthermore, new studies should evaluate whether vaccination, viral evolution, or reinfection alters the risk or profile of long-term persistent GI symptoms.

## Conclusion

In this study involving pre-vaccinated hospitalized COVID-19 survivors, the participants reported persistent GI symptoms two years after the acute infection. These symptoms were likely associated with altered systemic inflammatory markers and a distinct cytokine profile, particularly involving IL-6. The findings support the hypothesis that gastrointestinal manifestations of long COVID are consistent with an association with sustained immune dysregulation. Exploratory and hypothesis generating analysis suggested TNF-α may serve as early biomarker of persistent GI symptoms. We acknowledge that the findings, while robust within the Manaus cohort, may not extend to outpatient, non-hospitalized, or non-Brazilian populations due to differences in care and population structure, thus we recommend larger cohort and multi-centric studies be done to confirm the hypothesis of TNF-α as early biomarker of persistent GI symptoms.

It was observed that these GI symptoms frequently co-occurred with other long COVID features, suggesting a multisystemic nature of the condition. Given the burden of persistent gastrointestinal complaints in post-COVID patients and their potential impact on quality of life, targeted screening and follow-up should be integrated into long-term care strategies for COVID-19 survivors. In addition, further research is needed to understand the mechanisms driving these sequelae, especially in the context of evolving viral variants and widespread vaccination.

## Materials and methods

### Study design and setting

This was an exploratory, case-series study with a two-year follow-up involving the hospitalized, pre-vaccine COVID-19 patients from the Brazilian Amazon^52,53^. The study was conducted in Manaus, Brazil, a city severely affected during the first wave of the COVID-19 infections in 2020, creating a unique population-level exposure profile characterized by high transmission, high disease severity, and limited therapeutic options. The hospitalization of large numbers of patients during this period into the two randomized clinical trials enabled systematic clinical and laboratory assessments at the acute phase, prior to widespread vaccination and before later viral variants emerged. Studying survivors from Manaus therefore offered an opportunity to characterize long-term sequelae in individuals with severe, early pandemic SARS-CoV-2 infection, an infection context that differs meaningfully from cohorts exposed later in the pandemic. Moreover, the region’s demographic, socioeconomic, and healthcare characteristics differ from those in high-income settings where most long COVID studies have been conducted, providing needed representation of populations from the Brazilian Amazon and reducing geographic bias in the long COVID evidence base.

### Participants

The study participants were derived from previously described randomized clinical trials, that also involved their clinical, laboratory, and immunological assessments^52,53^. Briefly, eligible participants were adults (≥ 18 years) who were hospitalized with laboratory-confirmed SARS-CoV-2 infection between March and May 2020. COVID-19 diagnosis was established by real-time reverse transcription polymerase chain reaction (RT-PCR). Patients were included regardless of disease severity during hospitalization, although most cases were classified as severe or critical based on the severity criteria outlined in the National Institutes of Health Coronavirus Disease 2019 (COVID-19) Treatment Guidelines^54^. Long COVID in the participants was defined as per the WHO criteria, that is, the continuation or development of new symptoms 3 months after the initial SARS-CoV-2 infection and lasting for at least 2 months with no other explanation^55^. Exclusion criteria included pre-existing chronic gastrointestinal diseases (e.g., inflammatory bowel disease, colorectal cancer), pregnancy, or inability to complete long-term follow-up due to either death or refusal.

To put into context, the participants in this study were identified and enrolled between March to May 2020 during the pre-vaccination, first wave of COVID-19 infections in the city. They were later contacted between January and November 2022 for follow-up interviews, clinical evaluations and clinical/laboratory sample collections. The cytokine analysis was realized between January and June 2024.

### Sample size considerations

The study was exploratory and conducted in a post-hoc follow-up context where the attainable sample was constrained by participants’ availability and willingness to re-engage. Accordingly, we prioritized recruitment completeness over prespecified sample size targets. This approach is consistent with feasibility-based cohort designs used in early post-COVID investigations. Overall, the final sample size was determined by participant availability during post-discharge follow-up. Therefore, no a priori power calculation was undertaken; instead, the achieved sample informs effect size estimates for future powered studies.

### Clinical follow-up and data collection

Participants were followed at five distinct time points: day 1 (D1, admission), day 7 (D7), day 14 (D14), day 120 (D120, approximately 4 months), and day 720 (D720, 2 years after admission). At each visit, clinical information, medication use, and laboratory data were collected (some medical consultations were done via telemedicine). At the D720 follow-up visit, persistent gastrointestinal symptoms, were evaluated through structured clinical interviews. Participants were asked to report the presence and frequency of key gastrointestinal symptoms, including abdominal pain, gastroesophageal reflux, diarrhea, and dysphagia. Symptoms were classified as persistent when they were self-reported as occurring weekly during the period preceding the assessment. Although symptom severity was not quantified using a standardized scoring instrument, the weekly threshold was used as a proxy indicator of clinically relevant persistence over time. Sociodemographic data (age, sex, education, income), comorbidities (hypertension, diabetes, obesity), and COVID-19-related treatments (oxygen therapy, mechanical ventilation, corticosteroids, antivirals, and ACE inhibitors) were also recorded during follow-up.

### Outcome definition

The primary outcome was the presence of at least one gastrointestinal symptom at D720, considered a long-term GI sequela of COVID-19. Based on structured interviews at D720 informed by the Rome IV criteria for functional gastrointestinal disorders, participants were divided into two groups: those with persistent gastrointestinal symptoms at two years (GI group), and those without gastrointestinal symptoms at two years follow-up (nGI group). The gastrointestinal symptoms evaluated included abdominal pain, gastroesophageal reflux, diarrhea, dysphagia, or a combination of either. Secondary outcomes included associations with inflammatory markers and cytokine profiles.

### Laboratory measurements

Routine hematological and biochemical laboratory tests were performed at all time points at the Clinical Analysis Laboratory of Fundação de Medicina Tropical Dr. Heitor Vieira Dourado. These test panels included complete blood count, serum creatinine, liver enzymes (ALT, AST), ferritin, and C-reactive protein (CRP), platelet count, mean platelet volume (MPV), vitamins, and ions, among others.

Dosing of circulating cytokines in patient serum samples was performed using the Cytometric Bead Array (CBA) flow cytometry technique with the Human Inflammatory Cytokine Cytometric Bead Array (CBA) Kit (BD™ Human Inflammatory Cytokine Kit, BD Biosciences, San Jose, USA) following the manufacturer’s instructions. The BD™ CBA Kit allows for simultaneously and distinct fluorescence intensity detection of various soluble cytokines in the sample. Data acquisition was by flow cytometry (BD FACSCalibur™), and cytokine quantified using the FCAP-Array™ software to determine cytokine concentrations in pg/mL and their Mean Fluorescence Intensity (MFI). All samples collected on D1, D7, D14, D120, and D720 were stored at -80 °C until analysis. Pre-COVID-19 serum samples, from healthy donors obtained at Fundação Hospitalar de Hematologia e Hemoterapia do Amazonas (HEMOAM) biorepository were also included in the cytokine assays as controls. To ensure assay reproducibility and inter-batch consistency, cytokine assays were done in triplicate and equipment calibrated before running the assay.

To ensure analytical robustness of cytokine measurements, all samples from each participant were analyzed in the same assay batch to minimize inter-assay variability. Batch controls and internal reference standards provided by the BD™ Human Inflammatory Cytokine Kit were included in every run, and fluorescence calibration beads were used prior to acquisition to standardize detector sensitivity. The sample analysis was performed in triplicates. All flow cytometry acquisitions were performed by a single trained operator, and data processing followed a standardized gating strategy established by two independent analysts to ensure inter-operator consistency. Together, these controls support the reproducibility and reliability of the immunological data presented.

### Ethical consideration

The Research Ethics Committee of Fundação de Medicina Tropical Dr. Heitor Vieira Dourado under approval number CAAE 52378221.7.0000 and HEMOAM under approval number CAAE 56413316.9.0000.0009. All participants signed an informed consent form before inclusion and follow-up procedures. The study was conducted in accordance with the principles of the Declaration of Helsinki, the Brazilian National Health Council Resolution No. 466/2012, the International Conference on Harmonisation Good Clinical Practice (ICH-GCP R2) guidelines, and other applicable Brazilian regulatory and ethical standards.

### Statistical analysis

Missing clinical and laboratory data at follow-up timepoints were handled using complete-case analysis without imputation; participants with unavailable measurements for a given variable were excluded from that specific analysis but retained in other comparisons where data were available. Descriptive statistics were used to summarize demographic and clinical characteristics. Continuous variables were expressed as mean and standard deviation (SD) or median and interquartile range (IQR), according to data distribution (assessed via Shapiro–Wilk test). Categorical variables were presented as absolute and relative frequencies. The chi-square test or Fisher’s exact test was used for categorical variables. In addition, between-group comparisons were performed using Student’s t-test for normally distributed variables and Mann–Whitney U test for non-normally distributed data. Comparisons among the timepoints were performed using one-way ANOVA followed by the Tukey or Friedman tests followed by Dunn’s test; along with the paired t test or Wilcoxon matched-pairs signed-ranks test. Additionally, the Benjamini, Krieger and Yekutieli method for correcting for false discovery rate was done. Given the exploratory design and sample size, multivariable adjustment was also applied primarily to cytokine analyses to detect for confounders. Confounding was further minimized through baseline group comparability and exclusion of participants with pre-existing chronic gastrointestinal disease. A p-value < 0.05 was considered statistically significant. All statistical analyses were performed using GraphPad Prism, version 8.0.2, R software version 4.3.1 within RStudio (v2023.6).

### Biomarker signatures analysis

The biomarker signature analysis was carried out to assess the panoramic profile of serum mediators. This analysis was performed by classifying individuals as “low” or “high” producers of these immunological mediators. The classification was done using the global median of each molecule measured, calculated from the data of all patients (CG, GI and nGI), as described elsewhere^56–59^. The overall median, expressed in pg/mL, was used as the cutoff. Thus, the cutoffs used were: IL-1: 0.54 pg/mL; IL-6: 5.8 pg/mL; IL-10: 0.6 pg/mL; TNF: 2.61 pg/mL; IL-12: 1.09 pg/mL; IL-8: 17.16 pg/mL. The identification of potential biomarkers can be visualized in a Venn diagram, where molecules present in high concentrations exclusively in the GI group are highlighted. To complement the visualization, the colormap graph at the bottom of the image provides a scale of molecular production intensity. The color gradation, from gray/dark blue to light blue, denotes an increase in the production of the molecule, with the darker shades corresponding to the highest levels, that is, above the threshold of 50% of the global median.

### Biological networks

Correlation networks analysis was assembled to evaluate the multiple associations among the inflammatory cytokines in the patients and the controls. The association was determined by using the Spearman correlation coefficient and statistical significance was considered only if *p* < 0.05. After performing the correlation analysis, a database was created using Microsoft Excel. Then, significant correlations were compiled using the open source Cytoscape software, version 3.9.1. The biological Networks were constructed using circular layouts in which each cytokine is represented by a globular node, where the larger the nodule size, the greater the correlations established. The correlation indices (r) were used to categorize the correlation strength as negative (*r* < 0), moderate (0.36 ≥ *r* ≤ 0.68), and strong (*r* > 0.68), which were represented by connecting edges, as proposed by Taylor (1990)^60,61^. Cytoscape and Microsoft PowerPoint Software were used for graphics.

## Data Availability

All data generated or analyzed during for this study are included in this published article. Additional information or data regarding the study can be availed upon reasonable request.
